# Electricity Load and Price Forecasting Using Jaya-Long Short Term Memory (JLSTM) in Smart Grids

**DOI:** 10.3390/e22010010

**Published:** 2019-12-19

**Authors:** Rabiya Khalid, Nadeem Javaid, Fahad A. Al-zahrani, Khursheed Aurangzeb, Emad-ul-Haq Qazi, Tehreem Ashfaq

**Affiliations:** 1 Department of Computer Science, COMSATS University Islamabad, Islamabad 44000, Pakistan; rabiyakhalid672@gmail.com (R.K.); tehreemashfaq786@gmail.com (T.A.); 2 Computer Engineering Department, Umm AlQura University, Mecca 24381, Saudi Arabia; fayzahrani@uqu.edu.sa; 3 College of Computer and Information Sciences, King Saud University, Riyadh 11543, Saudi Arabia; kaurangzeb@ksu.edu.sa (K.A.); qulhaq@ksu.edu.sa (E.-u.-H.Q.)

**Keywords:** forecasting, energy management, data preprocessing, outliers, regression

## Abstract

In the smart grid (SG) environment, consumers are enabled to alter electricity consumption patterns in response to electricity prices and incentives. This results in prices that may differ from the initial price pattern. Electricity price and demand forecasting play a vital role in the reliability and sustainability of SG. Forecasting using big data has become a new hot research topic as a massive amount of data is being generated and stored in the SG environment. Electricity users, having advanced knowledge of prices and demand of electricity, can manage their load efficiently. In this paper, a recurrent neural network (RNN), long short term memory (LSTM), is used for electricity price and demand forecasting using big data. Researchers are working actively to propose new models of forecasting. These models contain a single input variable as well as multiple variables. From the literature, we observed that the use of multiple variables enhances the forecasting accuracy. Hence, our proposed model uses multiple variables as input and forecasts the future values of electricity demand and price. The hyperparameters of this algorithm are tuned using the Jaya optimization algorithm to improve the forecasting ability and increase the training mechanism of the model. Parameter tuning is necessary because the performance of a forecasting model depends on the values of these parameters. Selection of inappropriate values can result in inaccurate forecasting. So, integration of an optimization method improves the forecasting accuracy with minimum user efforts. For efficient forecasting, data is preprocessed and cleaned from missing values and outliers, using the z-score method. Furthermore, data is normalized before forecasting. The forecasting accuracy of the proposed model is evaluated using the root mean square error (RMSE) and mean absolute error (MAE). For a fair comparison, the proposed forecasting model is compared with univariate LSTM and support vector machine (SVM). The values of the performance metrics depict that the proposed model has higher accuracy than SVM and univariate LSTM.

## 1. Introduction

The emergence of smart grid (SG) technology has made the modern power system stable and increased its reliability. The communication between utility and consumers has proven to be beneficial for both parties. The former uses the information related to the demand and generates the effective incentive-based planes to control the pattern of demand up to some extent. Similarly, the latter receives the information related to price and incentives and alters the demand patterns to get the benefits in terms of low monetary cost. In spite of these positive aspects of the modern grid, there is still room for improvement. The main issues of the modern grid include improvement in energy efficiency, integration of renewable energy sources (RESs), and reduced emission of harmful gases which are a serious threat to the environment. These issues can be tackled by using the reliable and accurate information of future load demand, energy generation of RES, and suitable prices for upcoming intervals.

The integration of two-way communication in a power system generates data in a huge amount. In addition, the deployment of sensors and other similar components to monitor the environment also generate a massive amount of data daily. Reduced prices of storage mediums enabled utility companies to store this information on a daily basis. These databases have a variety of data which is growing rapidly with each passing day. Only collecting this big data is useless; if no useful information is being extracted from it then it is not beneficial. Big data analytics have become a hot topic for researchers who are trying to get the maximum benefit from it. Similarly, big data related to SG is being utilized by researchers to increase the efficiency, reliability, and stability of the grid along with making it environmentally friendly. In this regard, researchers have proposed forecasting models to predict the future demand [1], price [2,3,4], energy generation [5], and fault and anomalies of power systems [6] using big data. The implementation of these models can effectively increase the throughput of the modern grid.

The core aim of SG is the integration of information and communication technologies that enable electricity users and utility companies to interact and exchange information. Both market participants want to get maximum benefits by actively participating in energy management strategies. Their benefits highly depend on the accurate prediction of demand and the price of electricity. Researchers are actively working in this area and using big data with forecasting algorithms. There are two types of forecasting models: classical and intelligent models. The former include auto-regressive integrated moving average, dynamic regression, the generalized autoregressive conditional heteroskedastic model [7], and transfer function models [8]. These are well known classical methods for forecasting.

On the other hand, the latter are data-driven models which use historical data to train the model and, after training, predict the future values of the desired variables. These models include artificial neural networks (ANNs) and their variants, support vector machine (SVM) based on autoregressive moving average, etc. In this paper, a variant of ANN is implemented which is called long short term memory (LSTM) based recurrent neural network (RNN). This is a data-driven model which uses big data to train and map the input values to their respective output. It is a time series based model and usually works with a single value. In this paper, we have implemented this model with multiple input values to forecast the electricity price and demand. Moreover, the hyperparameters of this model are tuned using the Jaya optimization algorithm instead of the commonly used Adam optimizer.

Data preprocessing is also a very important phase of the electricity forecasting model. The data available for use is generally in raw form. In the preprocessing phase, it is cleaned and shaped according to the requirements of the forecasting model. The unprocessed and raw data leads the forecasting model to inaccurate predictions. A number of different data preprocessing techniques are available in the literature. In our paper, we have used only a selective number of input variables so we would not use any feature selection technique. Instead, we have used the z-score based method to eliminate the outliers from the data. Missing values are also removed and values of variables are normalized using a min-max scaler.

### 1.1. Related Work

The main objective of the study [4] was to predict electricity prices using big data. Electricity price forecasting plays a significant role in cost reduction in SG. Decision tree (DT) and ANNs are popular for forecasting. The DT face overfitting problem which means it is good for training the model but it does not perform well for prediction. On the other hand, the ANN suffers poor convergence as its convergence is not easily controllable. Moreover, these learning-based algorithms do not use big data for predictions, instead they use only price data. So, by using big data for prediction, there is still room for improvement in accuracy. To address the aforementioned issues, the authors used big data and an SVM based model for electricity price prediction. SVM has two major issues that affect its performance. The first one is computational time and the second is tuning of super parameters. The first issue is addressed by reducing the dimensions of data and applying feature selection and extraction techniques. In the first step, random forest, and relief forest algorithms are used to select the important features then KPCA is applied for feature extractions. For parameter tuning, the traditional method of grid search and cross-validation are used.

Two architectures were proposed for SG big data analytics along with a software application in [7]. This data was gathered from energy analyzers mounted at a suitable location in the SG system. The proposed software application was used for data analysis, monitoring, and collection. The main focus of this study was on peak clipping and load shifting. The performance of both frameworks was evaluated on the basis of latency, data rate, and network overhead. The results of both architectures depict that they are suitable and working efficiently on a small scale. However, the same architectures are extendable for the large scale. The representational state transfer (REST) interface is suitable for a relatively lower amount of analyzers. The integration of more analyzers in the network degrades the performance of the REST interface.

Traditionally, a data-driven or model-driven approach is used for dynamic event monitoring. However, the authors in [8] have proposed a framework for dynamic event monitoring which combines the features of both approaches for better and efficient performance. In the proposed framework, the functional components were formulated using phasor measurement unit. In the next step, the representative features were learned from each component. In the third and final step, neural network based classifiers were trained to detect and classify the data and events. Simulations were carried out using the IEEE 39-bus system for evaluation of the proposed framework. The results depict the efficient event detection and classification.

Forecasting of energy consumption was investigated for different event venues in the study [9]. Two forecasting methods, support vector regression (SVR) and neural networks, were used and their performance was compared on the basis of forecasting accuracy and mean absolute percentage error (MAPE) and cross validation error rate. Three scenarios were designed; one with fifteen-minute data, the second with one hour data, and the third with daily energy consumption data. Both methods were applied to these three scenarios and their performance was compared. The results show that both forecasting methods performed well in all three scenarios. However, the performance of neural networks, when applied to daily data, is better than SVR. Moreover, they concluded from performance evaluation that for the other two methods: fifteen minutes and hourly data, there is no dominance of one technique over another. Instead, both methods perform equally well. If we talk about the accuracy of peak consumption and total consumption then peak consumption was forecasted accurately by both models.

Authors in [10] presented a comprehensive study of energy management based on big data analytics. Data generation sources and their properties were discussed. Moreover, a data processing model is also proposed, where steps of big data analysis are elaborated and each step is briefly discussed. This study covers the energy management aspects, microgrid and assets management, and demand-side management. Additionally, the challenges of data-driven approaches in SG, related to data collection, infrastructure, storage, integration, processing manipulation, security, and privacy are discussed and their issues are highlighted.

The study [11] presents a detailed review of big data, its analysis methods, main issues, and challenges in the energy efficiency of buildings. A comparative analysis of big data research publications in energy with other disciplines is also presented in this paper. This comparison depicts the time-line of research publications on big data. The main issues of big data, taking out the required data in a limited time interval, and management of high dimensional data and limited processing capabilities of existing systems are highlighted. However, this paper does not suggest any suitable measure to address the issues of big data.

A data-driven approach was proposed to mine the energy consumption behavior of consumers over time in [12]. The frequent pattern mining technique was used to capture the association of appliances of a user. The energy consumption data, generated from smart meters, was used for this purpose and mining methods were applied as this data is easily available. The proposed mechanism is an incremental progressive method. The association of appliances changes frequently, so this model records these frequent changes and mines out the dependencies of appliances. They concluded that in order to alter the energy consumption pattern and reduce energy consumption, the “appliances of interest” play a very important role. Moreover, they also demonstrated in results that consumer’s energy consumption behavior is directly affected by the association of appliances.

Authors in [13] stated that energy consumption forecasting is beneficial for both building owners and utility. In this regard, the forecasting based on artificial intelligence and other conventional methods is reviewed. The conventional methods include stochastic time series and regression-based approaches. They concluded that the non-conventional methods are not efficient for non-linear data and they are also not flexible. On the other hand, the artificial intelligence-based methods discussed in this paper include ANN and SVM based methods and their variations. They stated that these methods have better performance than conventional methods and they perform quite well for data with non-linear patterns. Additionally, hybrid methods: hybrid SVM, hybrid neural networks and hybrid swarm-based methods are also discussed. These methods are proposed by researchers and proved to have better performance than originally available methods. They concluded that conventional methods are easy to develop but performance-wise artificial intelligence-based methods are better.

The study in [14] aims at the use of big data in the electrical power system for control, process, and protection purposes. As the volume of data is huge and increasing rapidly, traditional database tools are unable to process this data. Special tools are used to handle this type of data. So, in this study, three important aspects of big data usage, feature extraction, and integration and application, are discussed. Moreover, this paper outlines the application of big data in power systems for asset management, fault detection, operation planning, and distributed generation. Data management steps are also discussed and future research directions of big data implication in SG are outlined.

Authors in [15] discussed the application of IoT in SG. They have also considered the integration of RES in the system. They highlighted that IoT enabled SG generates a huge volume of data on a daily basis, which can be stored and used in the future. As this data is huge in volume, collected from heterogeneous sources, and increasing with the passage of time, it is referred to as big data. This big data can play a significant role in the operation, planning, and efficiency of future SG. Technologies used to store and process big data are also discussed in this paper. Moreover, an IoT framework for SG is also presented, which consists of three layers. The perception layer is used to collect data, the network layer transfers this data from source to destination using communication protocols, and the third layer is responsible for the definition of applications of this collected data, e.g., demand response, fault detection, demand profiling, the operation management of RES, etc.

The work of Jain et al. [16] explores the impact of temporal granularity (daily, hourly, and 10-min intervals) on the accuracy of electricity consumption forecasting. They achieved the best results with hourly intervals and monitoring by floor level. Jain et al. studied a residential building; this research is concerned with large commercial customers, specifically event-organizing venues. To handle large variations in energy consumption caused by events, they have included contextual information about future events such as event type and schedule. Moreover, in addition to consumption prediction, this work also includes peak demand prediction.

Authors in [17], proposed a short term load forecasting model for industrial applications. The proposed model is based on ANN and modified enhanced deferential evolutionary technique to improve the forecast accuracy. For feature extraction and network training, mutual information based techniques and a multivariate autoregressive model were used, respectively. The fast accurate short load forecast model is enabled via training to forecast the future load. Simulation results express that the proposed model provides 99.5% accurate predictions. However, the accuracy is improved by feeding the output of the forecasting module to the optimization module which takes more time to execute.

A real-time anomaly detection framework was proposed in [18]. This framework detects the abnormalities with the help of smart meter generated data. At each consumer’s side, the error count was measured and delivered to the system with which that user is linked. The error counts of each consumer were infused together to detect the anomalies in the system and acquire information about if an anomaly is affecting multiple customers or only a single customer. In this model, multiple levels of an anomaly are defined which are mutually exclusive and represented by M. A health vector was defined which represents the detected anomalies. The smart meter measurements are the error counts and each smart meter can have one anomaly at a time. The number of the error count is defined over a Poisson distribution. At the system level, an additional variable was used which represents the ratio of anomaly detected and N customers. Missing points in data are also determined and defined over a Bernoulli distribution. Missing points in data means whether the error count has reached the literal or reached it with latency.

A big data framework was proposed in [19] for SG related data. There are seven important stages of this framework: data generation, data acquisition, data storing, data querying, data analytics, data monitoring, and data processing. In this paper, authors have proposed a compact big data framework for SG, where they have discussed the open-source prevalent platform to tackle big data challenges and issues. In addition to the big data core components, the utilization of open-source state-of-the-art prevalent, Hadoop platform for addressing SGs big data challenges, are also discussed. The open-source tools are adopted, which provide an easy and cost-effective development environment for practicing engineering to develop similar tools for their demanding SG applications. Moreover, practical implementation of the framework, including necessary configuration and coding, and its application on real SG data are also presented. As data is gathered using a big data framework, this data can be used for forecasting by applying data mining techniques on available data. The forecasting can be beneficial for both utility and consumer. From the utility’s point of view, demand and energy consumption patterns of users can be predicted and energy generation and demand response programs can be implemented accordingly. From the user’s point of view, electricity cost can be predicted or electricity load information can be predicted and they can adjust their power usage accordingly.

Demand-side management is an important application in the future SG. In [20], the authors proposed a two-tier cloud-based demand-side management system that schedules the power consumed by customers in different regions and in microgrids so that both customer and utility company costs are optimized. As the optimization problem is addressed globally at the cloud level, so, the objective function is also global and is beneficial for both users and utility at the same time. The cloud-based nature of the proposed model reduces the cost of computation. The two-tier framework solves the network congestion and high computational time problems as well because each region has its own edge cloud for computation and users interact with it, only filtered data and necessary information related to optimization from edge clouds is forwarded to the core cloud.

A data-driven approach was used in [21] for anomaly detection in SG. In this work, the proposed architecture performed analysis and the final results were compared with the results obtained through random matrix theory (RMT). RMT is a purely mathematical model that is introduced in this paper to detect the anomalies along with the data-driven architecture. These methods are beneficial for both user and utility. The RMT uses a split window like a phenomenon to process data, so a group of consumers can share information and get the information in which they are interested in a small scale. Whereas, the utilities are big enough to control the groups and process their data.

The authors stated in [22], that the deployment of monitoring devices in SG is generating big data. The use of this big data plays a very important role in increasing the efficiency of the SG system. In this work, the focus of researchers is on the issues of the data collection side. Two limitations in the existing literature are highlighted. One is that data is extracted and used for a single task only, whereas, the same data can be used for multiple tasks, e.g., voltage stability and prediction of the load. The second limitation is that data is acquired from source nodes and delivered to the destination nodes for information extraction. Instead of this, the intermediate nodes should be able to extract information and forward only important information. To overcome these limitations, two algorithms were proposed. The first one determines the suitable number and location for power nodes (intermediate nodes) placement and the second algorithm calculates the in-path bandwidth saving ratio. The proposed big data framework is effective for the improvement of computational efficiency and saving the in-path bandwidth ratio. The emulator is designed in C++.

A forecasting model for electricity prices and load was proposed in [23]. This is a mixed forecasting model which uses an iterative neural network to forecast the future value of both electricity load and prices. From the results, they observed that the forecasting error, between forecasted values by model and real values of both electricity load and price, was very low. The forecasting problem is considered as an optimization problem, where, the objective is to reduce the forecasting error while forecasting the electricity load and prices simultaneously. As both variables are interdependent, the model uses values of both variables iteratively and does not include the values of known variables. Additionally, they claimed that this model can be applied to any real world scenario to forecast the future values of interdependent variables and the number of these variables can also vary and are not specific to two. The forecasting results of the proposed model were compared with existing techniques and it was evident that the forecasting accuracy of this model is better.

A load and price forecasting model was proposed in [24]. They stated that the price of electricity changes dynamically and its change depends on the difference between the available power and load demand at a particular time interval. To manage demand of electricity according to the available power, forecasting of both variables is of great importance. Two separate kernel methods are proposed to forecast the values of these variables. These forecasted values are then used to alter the load consumption pattern. The user interaction is minimal in this model and the user controls the values of only three variables, i.e., how much load he wants to curtail, how much electricity he wants to consume, and which load to shift. As a result, the user gets the monetary incentives in the form of low electricity consumption cost.

Authors in [25] proposed a two-staged short term load forecasting model that is price-dependent. They stated that the electricity prices were changed in real time according to the load consumption patterns. The consumers can alter their patterns according to the real time prices of electricity and minimize their electricity consumption cost. The proposed two-staged model first forecasts the load consumption without considering electricity prices. In the next stage, the price sensitivity is added to the forecasted values of the first stage. Fuzzy logic is used at this stage and to optimize the number of rules and parameters of the membership function automatically, a genetic algorithm is used. In this way, the forecasting values are improved and the forecasting error is minimized.

Another fuzzy logic based load prediction method is proposed in [26]. In this model, the electricity consumption was forecasted using a neural network using historical load data. After load prediction, the values were adjusted by incorporating the effect of electricity prices on load consumption. Fuzzy logic was used for this price adjustment process. From the simulation results, it can clearly be observed that the addition of the electricity price effect on load forecasting significantly improved the forecasting accuracy of the proposed model.

The real time pricing schemes greatly affect the power consumption patterns and cost of electricity of users. To manage load efficiently and reduce the cost, the knowledge of future values of electricity price is inevitable. To assist the users in this regard, a price forecasting model was proposed in [27]. A set of relevance vector machines is used to forecast the electricity price and later on these forecasted values are aggregated and their coefficients are determined. For an optimized ensemble method for price forecasting, a micro genetic algorithm was used. Moreover, the performance of the proposed model is compared with naive Bayes and auto regressive moving average in terms of mean absolute error (MAE). The results depict that the proposed ensemble method has the highest forecasting accuracy.

### 1.2. Motivation

The core aims of SG are to create a balance between demand and supply of electricity and to reduce peaks in electricity demand. Consumers play a very important role in achieving these goals by participating in demand response programs. The key factor that makes the users take part in such activities is the use of dynamic pricing. The user tries to shift their load to where the price is low. On the other hand, electricity demand estimation for the next intervals helps the utility to create a balance between electricity demand and generation. So, electricity price and demand forecasting have significant importance in modern SG for both users and utility. In the literature, DT and other learning algorithms are popular for forecasting [18,19]. The DT faces the overfitting problem, which means that it is good for training the model but it does not perform well for prediction [18]. Moreover, learning-based algorithms do not use big data for predictions instead they use only price data. So, by using big data for prediction there is still room for improvement in accuracy.

With the fast emergence of SG, data is being acquired from a number of different devices on a large scale and stored as big data. This data can be utilized for making decisions related to the future planning of the energy generation sources and to make the SG more reliable, efficient, and sustainable. Electricity price and demand forecasting can be the major applications of big data in SG. Both electricity price and demand are influenced by a number of factors, for example price is influenced by fuel price, electricity demand, renewable energy supply, etc., and demand is affected by electricity price, weather conditions, users’ behavior, etc., their values change every hour with the change in these parameters. So, the utilization of large historical data can result in a more accurate prediction.

### 1.3. Contributions

This paper is the extension of [28]. To sum up this work, the main contributions are listed as follows:

- For electricity demand and price prediction, a multi-variable forecasting model Jaya-LSTM is proposed.
- The data set is preprocessed and cleaned before passing it on to the forecasting model. Outliers and missing values are removed and the values of input variables are normalized to make them comparable.
- To increase the forecasting accuracy of the model and get the best forecasted values, hyperparameters are tuned using the Jaya optimization algorithm. This is used as it is simple to apply and does not require deep knowledge and great optimization performance.
- The proposed model is tested on two separate data sets of price and demand of electricity. For evaluation, we have compared this model with SVR and classic LSTM. The performance is measured on the basis of two performance metrics, i.e., MAE and MAPE.

### 1.4. Organization of Paper

This paper is organized as follows: Section 2 contains the problem statement of our work. In Section 3, the proposed forecasting model is described along with its system model. Simulation results are discussed in Section 4. Section 5 concludes the paper and highlights the main findings.

## 2. Problem Statement

In this paper, our main objective is to predict electricity prices and demand using big data. Electricity price forecasting plays a significant role in cost reduction and demand forecasting helps to maintain the balance between electricity demand and supply in SG. In [4], differential evolution based SVM is used for price forecasting. A comparison with other existing algorithms, DTs and naive Bayes, is also provided. Here, the performance of the proposed model is better than these two algorithms (DT and naive Bayes). However, the performance of SVM is greatly influenced by the complexity, outliers, and size of a dataset. In the case of a complex dataset with more outliers, the forecasting becomes less accurate. Moreover, a huge dataset increases the computational complexity of SVM. In [29], a RNN based LSTM algorithm is used for electricity demand forecasting. In this work, only electricity demand data is taken into consideration for forecasting. However, the demand for electricity depends on different factors, e.g., the price of electricity, users’ behavior, weather condition, etc. So, to address the aforementioned challenges and issues, we have used multi-variant LSTM for both electricity price and demand forecasting. The neural networks work well with large and complex data sets and do not necessarily require feature engineering which makes them highly flexible. Moreover, the hyperparameters, number of epoch, batch size and window size, are tuned using the Jaya optimization algorithm and this forecasting model is named Jaya-LSTM (JLSTM). The use of JLSTM instead of LSTM enables the system to accommodate multiple input features along with tuned values of its hyperparameters according to the data set which increases the accuracy of forecasting.

## 3. Proposed Solution

**Figure 1:**
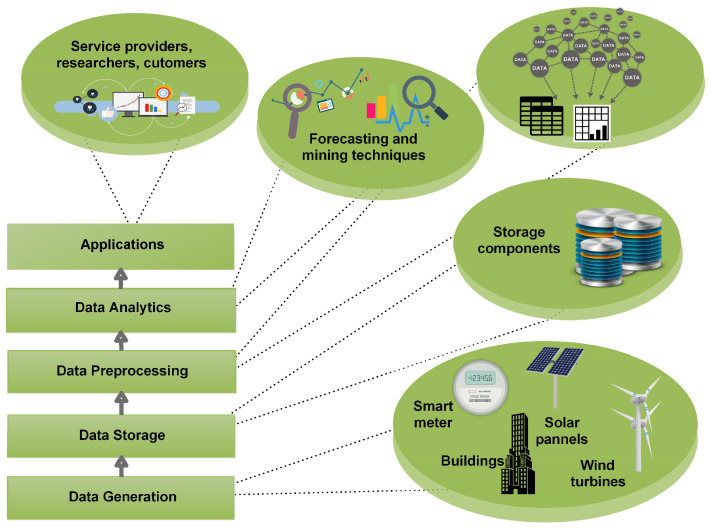
Framework of bigdata.

**Figure 2:**
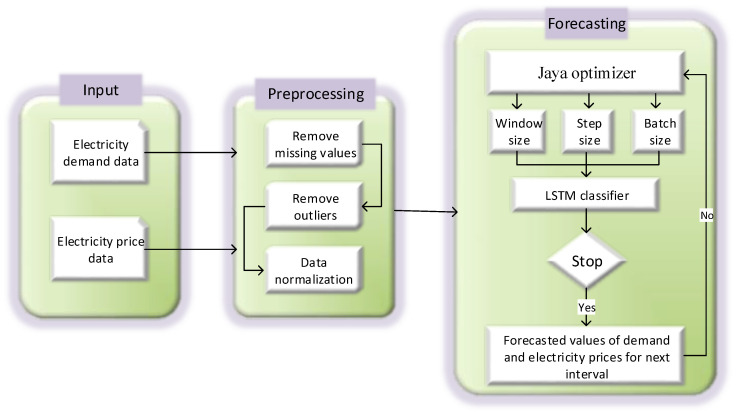
Flow diagram of the proposed JLSTM model.

Figure 1 shows the framework of big data. The first step is data generation. Data generated from different resources e.g., microgrids, smart buildings, smart meters, electric vehicles, factories, etc., is collected. This data is in raw form and generated in a tremendous amount. The second major step is the storage of this data for future use. This data can be stored in warehouses or services of clouds can be acquired for storage. When data is retrieved for use, preprocessing is a necessary step. In this step, big data is cleaned, important features are extracted, additional and ambiguous data is filtered out, and data is converted in tabular form to apply different analytical methods. In the next step, forecasting and data mining techniques are applied to extract the required information. After information extraction, it is used to make important decisions in different fields. In our scenario, we have applied JLSTM in the data analytics step and forecasted the demand and price of electricity. The information of our forecasted parameters can be used on both the user’s side as well as the utility side. A user can use this data in energy management controllers to efficiently manage its load, reduce the monetary cost of electricity, increase comfort, and reduce PAR. Whereas, the utility can use this information to keep the balance between demand and supply and make other important decisions related to electricity generation. Figure 2 shows the flow diagram of our proposed forecasting model. The phases of this model are discussed in detail.

### 3.1. Input Data

In this phase, the data required for forecasting is collected. In our model, two data sets are used i.e., electricity demand and price data [30]. The data set contains hourly data. We have used publicly available data for the sake of reproducibility of the results. In the literature, a number of different parameters are used as input variables for both price and demand forecasting. The increased number of input variables makes the model complex and requires more computational time as well as more hardware resources for efficient forecasting. There also exist studies having only one input variable for forecasting. Such models use the historical values of input variable and predict its values for the future. So, in our model we have used only most relevant variables which fulfill the minimum requirements of the model. After selection of data, the next phase is data preprocessing.

We suppose the following is the input data for price which is given as input to the forecasting model:

$$P=\left[\begin{array}{ccccc} p(I_{1}D_{1}) & p(I_{2}D_{1}) & p(I_{3}D_{1}) & \ldots & p(I_{n}D_{1})\\ p(I_{1}D_{2}) & p(I_{2}D_{2}) & p(I_{3}D_{2}) & \ldots & p(I_{n}D_{2})\\ p(I_{1}D_{3}) & p(I_{2}D_{3}) & p(I_{3}D_{3}) & \ldots & p(I_{n}D_{3})\\ p(I_{1}D_{4}) & p(I_{2}D_{4}) & p(I_{3}D_{4}) & \ldots & p(I_{n}D_{4})\\ . & . & . & . & .\\ . & . & . & . & .\\ . & . & . & . & .\\ p(I_{1}D_{n}) & p(I_{2}D_{m}) & p(I_{3}D_{m}) & \ldots & p(I_{n}D_{m}) \end{array}\right]$$

where, *D* represents days and *I* represents the forecasting intervals of those days. This matrix has nXm size, where, $D_{1}$ represents the first day of the historical data and $D_{m}$ represents the mth day. Similarly, $I_{1}$ represents the first interval of the day and $I_{n}$ represents the last nth interval of that day. Here, $p(I_{1}D_{1})$ represents the electricity price related data at first interval of the first day of data sample. $p(I_{n}D_{1})$ represents the nth interval of first day. Similarly, $p(I_{n}D_{m})$ represents the nth interval of the mth day in a data set. The input data for the demand is same as the price data and it also represents data according to the days and intervals. The input data set for demand can be represented as follows:

$$d=\left[\begin{array}{ccccc} dm(I_{1}D_{1}) & dm(I_{2}D_{1}) & dm(I_{3}D_{1}) & \ldots & dm(I_{n}D_{1})\\ dm(I_{1}D_{2}) & dm(I_{2}D_{2}) & dm(I_{3}D_{2}) & \ldots & dm(I_{n}D_{2})\\ dm(I_{1}D_{3}) & dm(I_{2}D_{3}) & dm(I_{3}D_{3}) & \ldots & dm(I_{n}D_{3})\\ dm(I_{1}D_{4}) & dm(I_{2}D_{4}) & dm(I_{3}D_{4}) & \ldots & dm(I_{n}D_{4})\\ . & . & . & . & .\\ . & . & . & . & .\\ . & . & . & . & .\\ dm(I_{1}D_{n}) & dm(I_{2}D_{m}) & dm(I_{3}D_{m}) & \ldots & dm(I_{n}D_{m}) \end{array}\right].$$

### 3.2. Data Preprocessing

Data preprocessing is a data mining technique used to transform raw data into a meaningful and understandable format. The data acquired from the real world lacks consistency, contains errors, is incomplete, and contains no evident behaviors. So, before using this data, we need to transform it into an understandable format. For this purpose, data preprocessing techniques are applied on the raw data. In the preprocessing phase, data goes through a series of data cleaning and optimization steps:

- Data cleaning is the first step. The missing values are filled or removed from the data set along with smoothing noise and inconsistency of data.
- In data integration, conflicts between data are resolved while integrating the data of different representations.
- Data transformation is also an important step, where the values of variables are normalized between a common interval to make them comparable.
- In the data reduction step, data is reduced by excluding irrelevant and duplicate information.

#### 3.2.1. Remove Missing Values

Removing missing values is an important step in data preprocessing. The missing data can reduce forecasting accuracy and lead to a misleading conclusion. Therefore, it is important to handle the missing values in data before forecasting. Several methods are available for this purpose. Removing rows of data is one of them. As the name suggests rows with missing values are excluded from the dataset. Another method is to take the mean of all values and fill the missing values. Forecasting algorithms are also used to predict the missing values of the data using the information of the present values. To select the best method, researchers go from simpler methods to the complex ones. In our paper, we have used the first method as the number of missing values in our data set is very low. So instead of increasing the computational overhead we simply removed the rows with missing values using python’s pandas library command.

#### 3.2.2. Remove Outliers

The outliers are those values in a data set which vary significantly from other values in a dataset. In SG electricity demand data, these outliers could be the result of public holidays like Christmas day or in case of some special events, where the demand of energy is higher than normal days. During the forecasting, it is better to exclude such data from the data sets, as we forecast the energy consumption for the normal days. Data values observed during these days can lead the forecasting model to a poorly trained model. To remove such values, Z-score is the commonly used technique by researchers [31].

(1) z=(x−μ)/σ.

Equation (1) is used to compute the z-score, where μ represents the average and σ is the standard deviation from the mean. This value is calculated for every sample in the data. To remove the outliers, a threshold is set. There is no hard rule to select the threshold as there is a trade-off in removing outliers. As data from the dataset means loss of information and keeping the outliers misleads the forecasted values. We need to balance it. The value of selected outliers differs with the dataset and scenarios.

#### 3.2.3. Normalize Data

In ANN we need to normalize data before passing it on to the forecasting algorithm for prediction. In a data set, the values of different variables do not lie in the same range. To make these values comparable, we normalize their values. The unnormalized data can result in an ill-conditioned network. Normalization of data also plays a very important role in the stable convergence of weights and other hyperparameters. To normalize our data, we have used a min-max scaler. The range of data values is set between 0 and 1.

(2) $$x_{norm}=\left(x-x_{min}\right)/\left(x_{max}-x_{min}\right).$$

Equation (2) is used to normalize the values of the dataset. Here, $x_{norm}$ represents the normalized values, *x* is the initial value in data set, $x_{max}$ represents the maximum of the whole data, and $x_{min}$ is its minimum value.

### 3.3. Forecasting Algorithm

After the completion of the data preprocessing phase, the data is now ready to pass on to the forecasting phase. In our paper, we have proposed JLSTM. The working and basic architecture of JLSTM is the same as LSTM, the only difference is that in JLSTM, multiple variables are used and hyperparameters are tuned using the Jaya optimization algorithm. An LSTM model was proposed by Hochereiter in 1997 [32] and it is a variation of RNN. This is a popular time series forecasting model which is applied in various fields and efficiently uses the data with log-term dependency for forecasting. As it is a variation of RNN, its internal architecture is similar to the RNN.

The internal connection of layers enables the signals to move forward and backward which makes them a suitable choice for time series forecasting. The RNN based forecasting models mine the rules and predict the future values of the data. It is made possible by the backpropagation feature which also plays a very important part in updating the weights. Existing RNN faces the gradient vanishing limitation, which is overcome by LSTM. It efficiently deals with data with long dependencies. It mainly has three gates i.e., input gate, output gate, and forgetting gate. The addition of the forgetting gate in LSTM is the reason for the mitigation of gradient problem [14]. Another noteworthy addition in LSTM is the memory cell. The following equations are used by LSTM as the activation function for all three gates [33]:

(3) $$f_{t}=\sigma \left(W_{f}\times \left[h_{t-1}+x_{t}\right]+b_{f}\right),$$

(4) $$i_{t}=\sigma \left(W_{i}\times \left[h_{t-1}+x_{t}\right]+b_{i}\right),$$

(5) $$o_{t}=\sigma \left(W_{o}\times \left[h_{t-1}+x_{t}\right]+b_{o}\right).$$

The Equations (3)–(5) represent the activation function for the forgetting gate, input gate, and output gate respectively. They use sigmoid function σ, where *W* represents the weight, *x* is the input, *h* is the hidden state, and *B* represents the bias vector in each equation [33].

(6) $$\varsigma_{t}=tanh\left(W_{\varsigma }\times \left[h_{t-1}+x_{t}\right]+b_{\varsigma }\right).$$

(7) $$c_{t}=f_{t}\times c_{t-1}+i_{t}\times \varsigma_{t}.$$

(8) $$h_{t}=o_{t}\times tanh\left(c_{t}\right).$$

In Equation (6), a new candidate value is computed. A new cell state is reckoned in Equation (7). In this equation, the old cell state $c_{te1}$ is multiplied by forgetting gate $f_{t}$ and in this way the information of the old cell is discarded. The input gate is multiplied by candidate value $\varsigma_{t}$ and both values are added in the end. Equation (8) computes the value of required information which would be used in the next step as a hidden state. Batch size is an important parameter to train the RNNs. It represents the number of training samples used at the time of the weighing updation of the network. The following equations are used to create the batch [33]:

(9) $$\begin{array}{c} x\_Batch(i)=x\_train(r),x\_train(r+(seq\_len-n)),\ldots ,x\_train(r+seq\_len), \end{array}$$

(10) $$\begin{array}{c} y\_Batch(i)=y\_train(r),y\_train(r+(seq\_len-n)),\ldots ,y\_train(r+seq\_len), \end{array}$$

where

r=rand(a,b).

In Equations (9) and (10), *i* represents the ith samples of batch, and *r* is a random number generated between the range *a* and *b* at each iteration *i*. It acts as an index value to choose the sample for the batch from the dataset. where seq_len represents the number of time steps.

In forecasting algorithms, the accuracy is greatly affected by their hyper parameters. So, the values of these parameters should be chosen carefully. In this paper, we have optimized the values of batch size, epochs and window size. In the existing literature, researchers prefer nature inspired algorithms due to their great efficiency. However, these algorithms are probabilistic and need the careful selection of their tuning parameters e.g., size of the population, generation, and elite. If their respective parameters are not tuned optimally, they fall into either local optima or require more computational time. Owing to these issues, the Jaya optimization algorithm is used in this work for LSTM optimization. It is a newly proposed algorithm by Rao in 2016 [34]. This algorithm does not have any tuning parameters and its implementation is also simple.

In this work, three design variables are defined as only three parameters need to be tuned i.e., batch size, epochs, and window size. Population size is considered equal to 50 (the algorithm is run several times to get the optimal population size). The most important step is the definition of the objective function as the solutions are evaluated on the basis of this function. Here, our objective is to minimize the forecasting error of the model. Equation of mean absolute error (MAE) is used to compute the error which is defined as follows:

(11) $$obj=min(\frac{\sum_{i=1}^{n}\left|pr_{i}-Ac_{i}\right|}{n}),$$

where, *n* is equal to the the total number of predicted values, $pr_{i}$ is the ith predicted value and $Ac_{i}$ is the actual value.

After the evaluation of population, two solutions are selected: best and worst. The values of these solutions are then used to update the current population and the new generation is produced. The following equation is used for this purpose [34]:

(12) $$\begin{array}{c} X_{x,y,z}^{{}^{\prime }}=X_{x,y,z}+r_{1,b.c}(X_{x,best,z}-|X_{x,y,z}\left|\right)-r_{2,j,i}(X_{x,worst,z}-|X_{x,y,z}\left|\right). \end{array}$$

Suppose $X_{x,y,z}$ is an individual of the population which needs to be updated. Equation (12) is used to update this solution. Here, $X_{x,y,z}^{{}^{\prime }}$ is the updated solution and $r_{1,b.c}$ and $r_{2,j,i}$ are the two random numbers multiplied to the best and worst solutions of the current population represented by x,best,z and $X_{x,worst,z}$, respectively. After multiplication, absolute of $X_{x,y,z}$ is subtracted from both values and then subtraction is performed to get the next updated value. After getting the optimized values, these values are forwarded to the forecasting model.

## 4. Simulation Results

Our forecasting model is applied on two data sets of demand and price [30]. The simulations are carried out on a PC with Intel Core i3 CPU with 4 GB of RAM and a 64-bit operating system in Python version 3.6. The simulation results of the forecasted values of both demand and price of electricity are discussed in this section.

**Figure 3:**
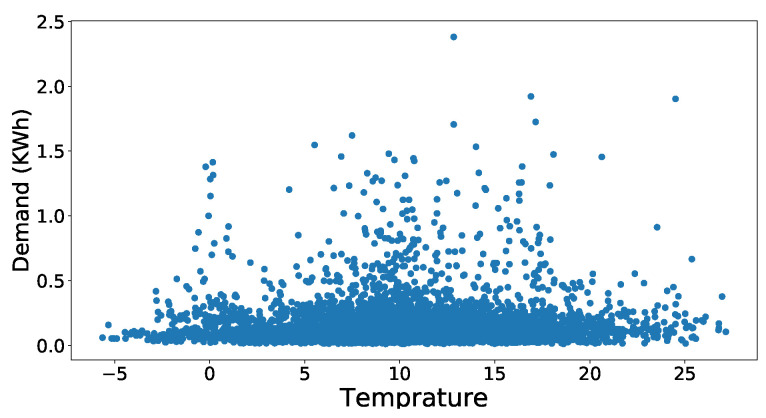
Dataset of demand with outliers.

**Figure 4:**
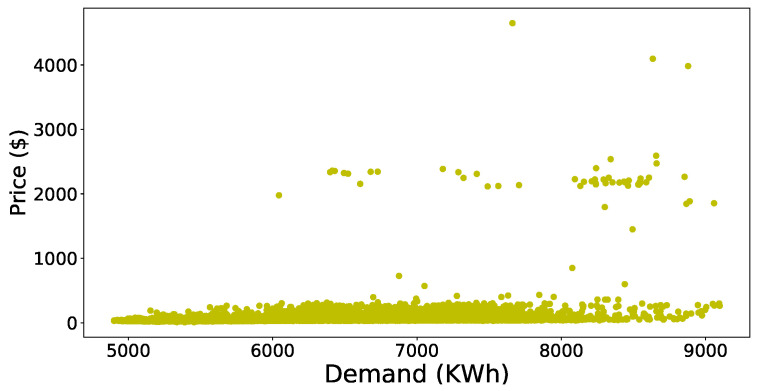
Dataset of price with outliers.

**Figure 5:**
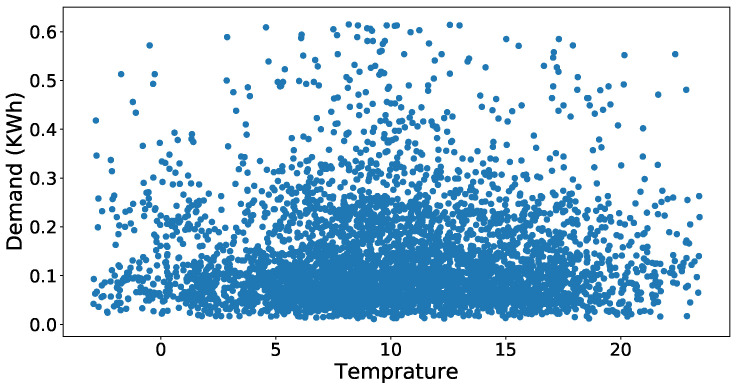
Dataset of demand without outliers.

**Figure 6:**
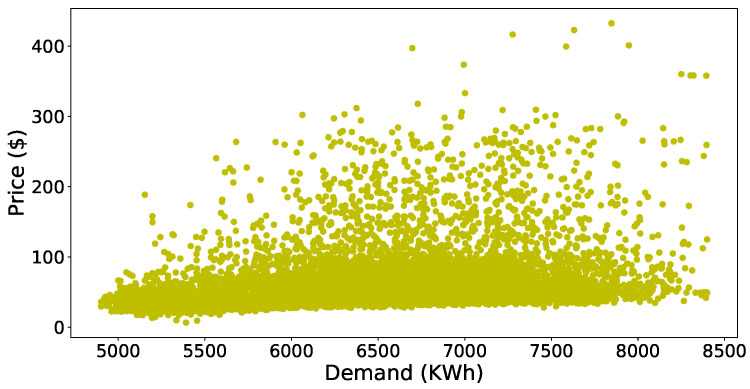
Dataset of price without outliers.

First of all, we will discuss the simulation results of the data preprocessing phase and then forecasted accuracy of the JLSTM for electricity demand and prices. Figure 3 shows the scatter plot of the demand dataset. It is evident from the figure that the outliers are present in the data set. The data points present in the upper half of the scatter plot have more distance between them as compared to the points present in the lower half. Similarly, the scatter plot of price data is shown in Figure 4. This plot also shows the presence of outliers in the price data set. The number of outliers in the price data set seem more as compared to the demand. However, it is not necessarily correct; we apply some mathematical methods to compute the values of every point and then identify the outliers on the basis of those values. Here we have applied the z-score to compute the values of these points. After computing the values, we need to set a threshold to remove these outliers. The selection of the threshold should not be very strict as it may result in more information loss which results in a low accuracy of prediction. So, to balance the trade-off between loss of information and outliers we have chosen a loose threshold value. Figure 5 shows the data points after the outliers are removed from electricity demand data. It depicts the outliers that are successfully removed, and the distance between the data points is not abnormal. However, the distance between the points present in the upper half of the plot is still greater as compared to the lower half but this is justifiable as we need to take care of the trade-off also. Similarly, Figure 6 shows the scatter plot of the price data after removing the outliers from it.

**Figure 7:**
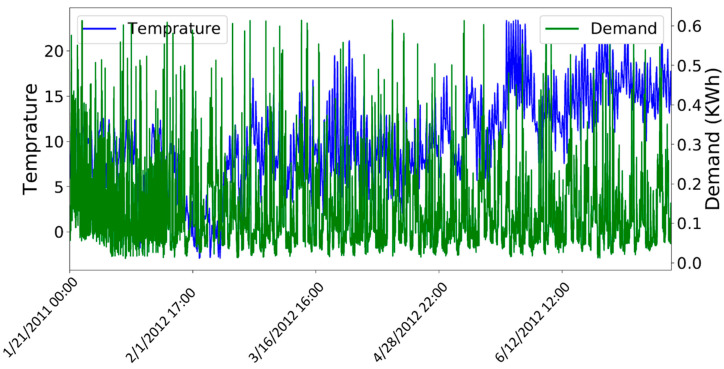
Dataset of demand before normalization.

**Figure 8:**
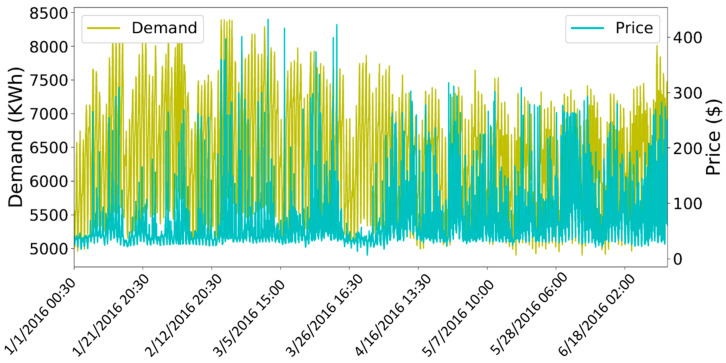
Dataset of price before normalization.

**Figure 9:**
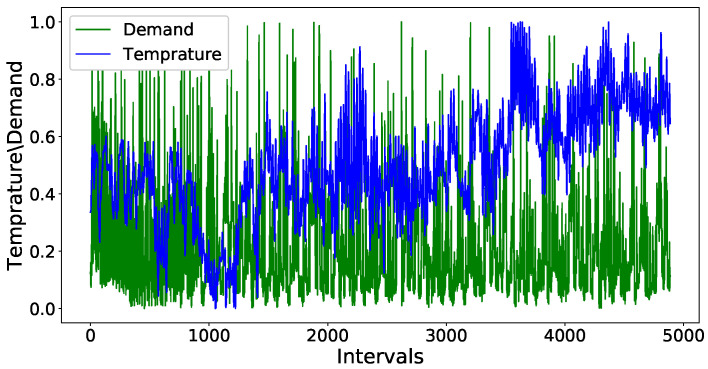
Normalized dataset of demand.

**Figure 10:**
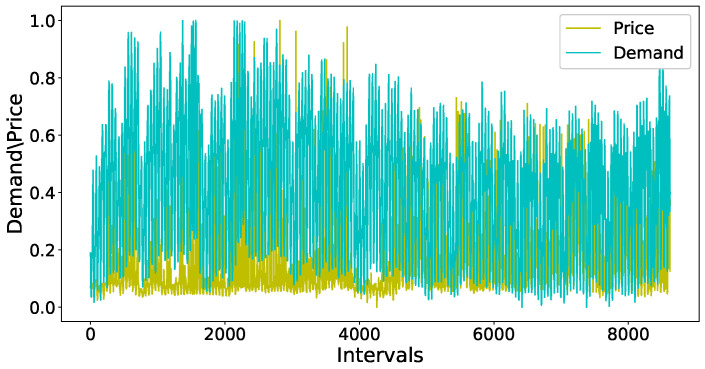
Normalized dataset of price.

After removing the outliers, the next step is to normalize the values of variables in the dataset between a common range. Normalization is important as the variables which are not scaled on a common interval are difficult to compare and may lead to an ill-trained forecasting model. Figure 7 shows the original values of both the demand of electricity and temperature variables of the electricity demand dataset. The left side of the y-axis shows the range of values for temperature and the right y-axis contains the tick labels for the demand variable. Figure 8 shows the unnormalized values of price data set. The left y-axis contains the tick labels for the original values of electricity demand and the right y-axis represents the tick labels for the unnormalized price. A min-max scaler is used to normalize the values of both datasets between interval 0 and 1. Figure 9 and Figure 10 show the normalized values of all four input variables.

**Figure 11:**
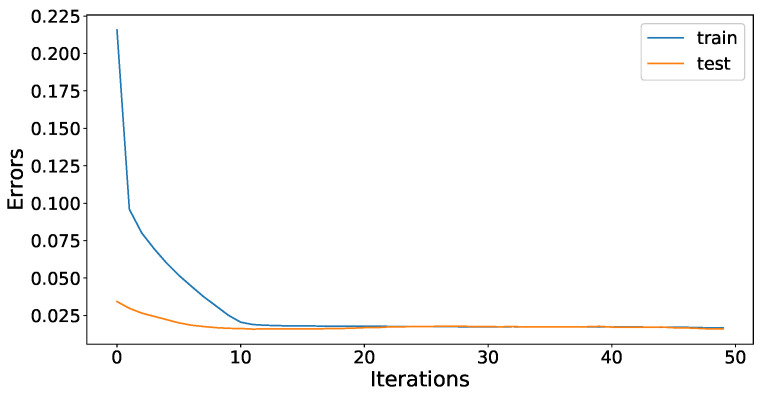
JLSTM model training error for demand.

**Figure 12:**
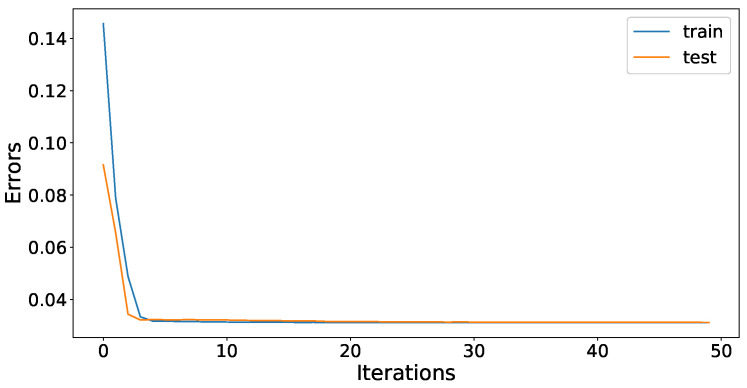
JLSTM model training error for price.

The cleaned and processed data is now ready to be used by the forecasting model. The JLSTM is applied on the data for forecasting. The hyperparameters of the JLSTM are tuned by using the Jaya optimization algorithm and the Adom optimizer is used as a local optimizer inside the model. The value of max-iterations is set to 50. The optimizer tunes the parameters and increases the forecasting accuracy of the model. Figure 11 and Figure 12 show the history of the model training for demand and price data, respectively. The accuracy of the model is evaluated using root mean square error (RMSE). Moreover, the test and train accuracy improves significantly during the initial iteration and the rate of accuracy improvement decreases with each iteration. As the number of iterations reach fifty, the test and train error for both electricity demand and price becomes constant. This is the point when we terminate our training phase of the forecasting algorithm and the model is now fully trained to make predictions.

**Figure 13:**
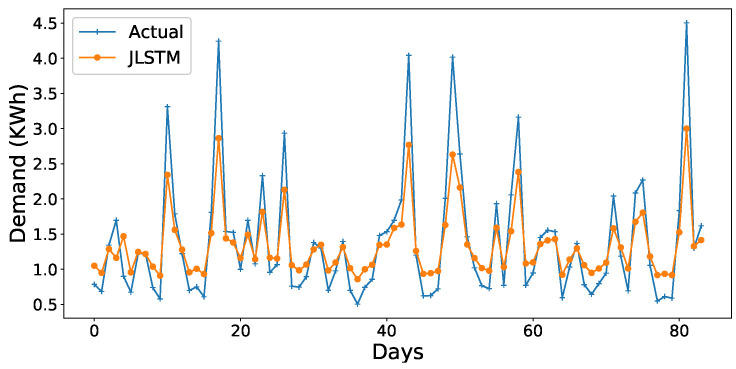
Forecast of demand for three months.

**Figure 14:**
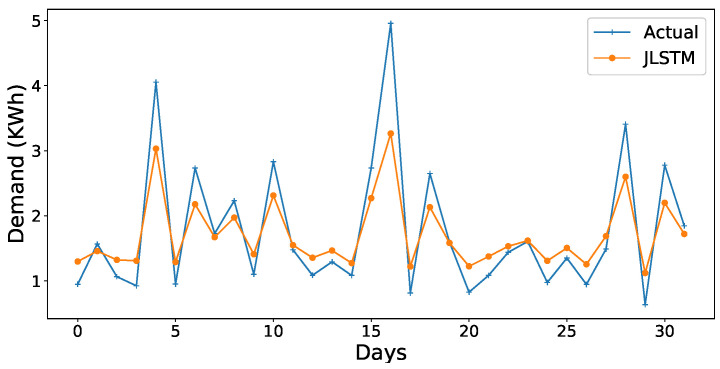
Forecast of demand for one month.

**Figure 15:**
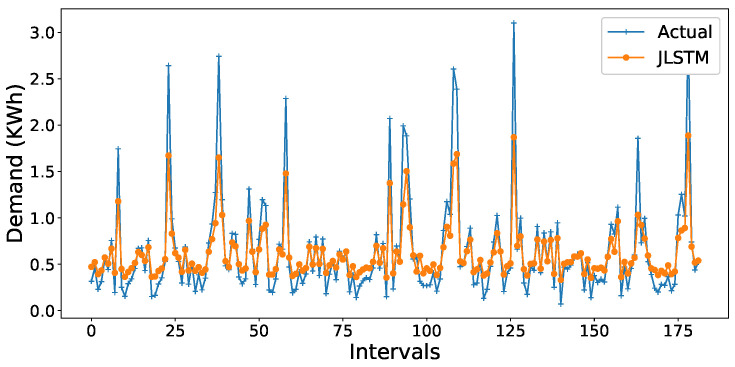
Forecast of demand for one week.

**Figure 16:**
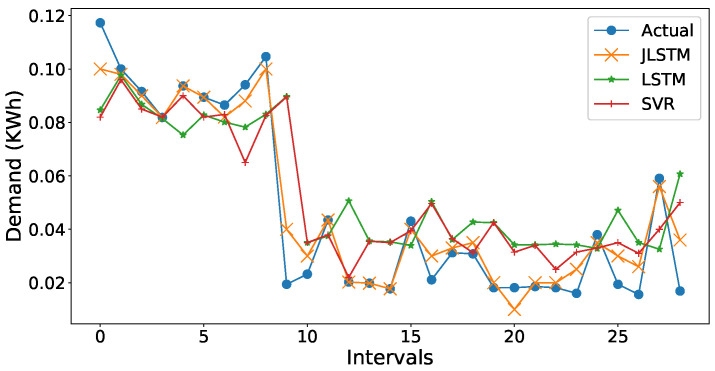
Comparison of forecasting models for demand prediction.

**Figure 17:**
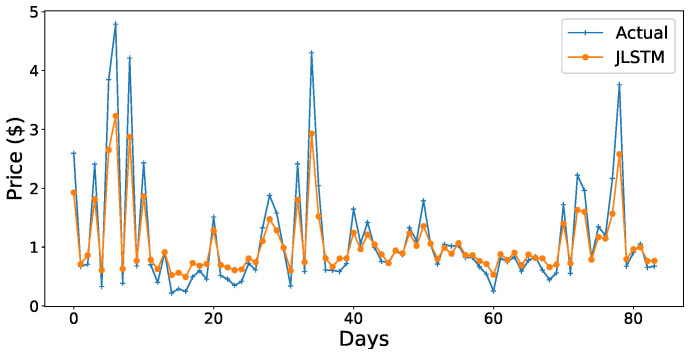
Forecast of price for three months.

**Figure 18:**
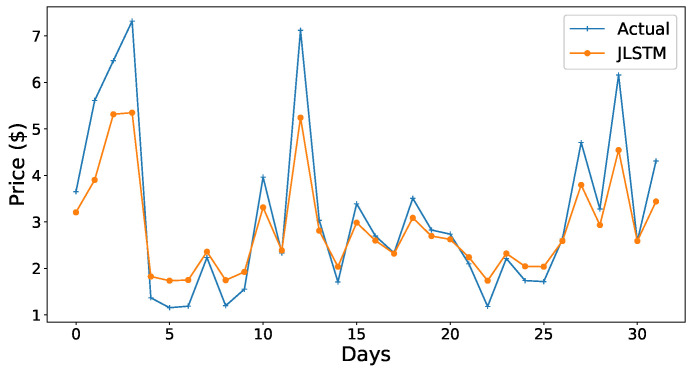
Forecast of price for one month.

**Figure 19:**
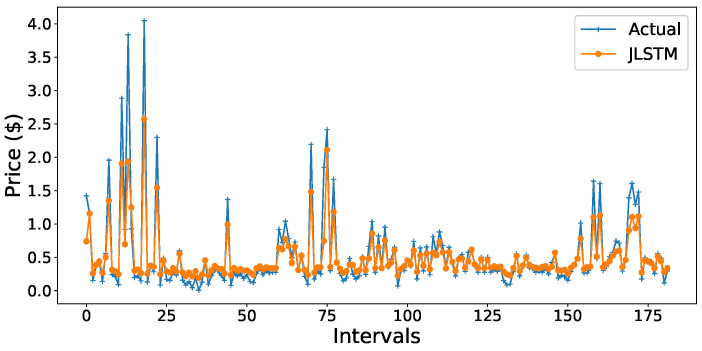
Forecast of price for one week.

The forecasting interval is of one hour. We have forecasted the values of both the demand and price of electricity for one week, one month, and three months. Figure 13, Figure 14, Figure 15, Figure 16, Figure 17, Figure 18 and Figure 19 depict the forecasting results of JLSTM for three months, one month, and one week for both the demand and price of electricity, respectively. To evaluate the accuracy of JLSTM, RMSE and MAE are used as performance metrics. The RMSE for demand and price is equal to 0.02 and 0.04 respectively. The MAE for the forecasted values of demand and price is 0.1 and 0.4 respectively. The performance of JLSTM is also compared with classic LSTM and SVM. The results of this comparison are depicted in Table 1. The values of both RMSE and MAE represent that the performance of the JLSTM is the best.

**Figure 20:**
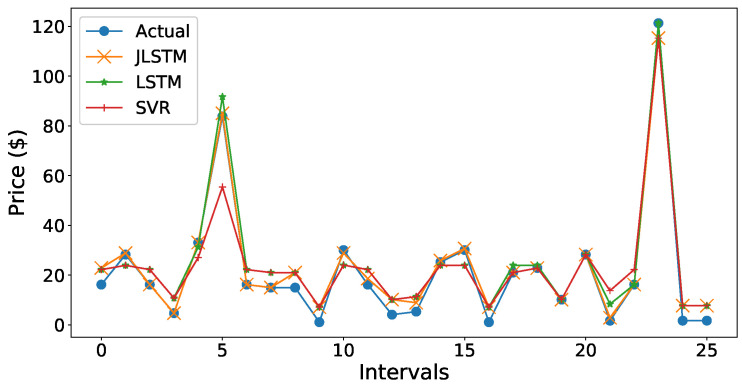
Comparison of forecasting models for price prediction.

**Figure 21:**
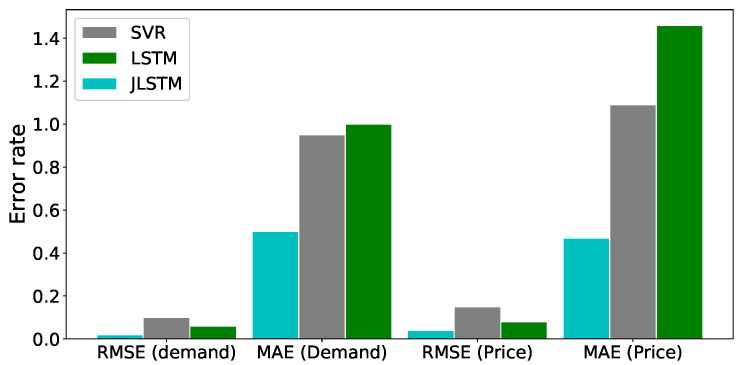
Comparison of forecasting error.

The comparison of forecasted values by JLSTM, LSTM, and SVR is graphically illustrated in Figure 16 and Figure 20. We can be observe from the figures that the prediction plot of JLSTM is closer to the plot of actual values in both price and demand cases. If we observe both the figures, they depict that the performance of LSTM is better than SVR. The comparative error rate is illustrated in Figure 21. This figure depicts that the error difference between SVR and LSTM is low as compared to the error difference with JLSTM as it achieves the highest forecasting accuracy.

## 5. Conclusions

Electricity load and demand forecasting are inviable for the stability and efficiency of modern SG. Knowledge of future values of electricity demand and price can significantly improve the efficiency of these systems. In this paper, the JLSTM forecasting model is proposed for the forecasting of electricity demand and price. Data preprocessing steps are also included in the proposed forecasting model. In the first phase, outliers are detected and removed from the data set using z-score. The values of the data set are scaled on the common interval to make the variables comparable. Missing values are also removed from the dataset. The cleaned data is then passed to the forecasting model to predict the electricity demand and price values for one week, one month, and three months. The Jaya algorithm and Adam optimizers are used to tune the hyperparameters of JLSTM. The former is used to optimize the batch size, the number of epoch and window size, whereas, the latter is used as an internal optimizer of the model. Forecasting accuracy of the model is evaluated using RMSE and MAE. Moreover, the performance of JLSTM is also compared with SVM and classical LSTM. The RMSE and MAE of SVM are 0.10 and 0.95, respectively, for demand forecasting. Whereas, LSTM has slightly high MAE, i.e., 1.4 but its RMSE is low, i.e., 0.06. Our forecasting model outperforms both forecasting models by achieving the lowest RMSE and MAE values, i.e., 0.02 and 0.1, respectively. For electricity price forecasting, SVM performs better than LSTM in terms of MAE as its MAE value is equal to 1.09 and LSTM has MAE value equal to 1.56. JLSTM beats both forecasting algorithm by achieving the minimum MAE value, i.e., 0.47. In case of price’s RMSE, once gain JLSTM has the lowest value, i.e., 0.04, and LSTM is second best having RMSE equal to 0.08, whereas, SVM has the highest RMSE, i.e., 0.15. The results of the performance metrics clearly show the best performance of JLSTM for both demand and price forecasting.

## Figures and Tables

**Table 1:** Error in forecasting accuracy.

	JLSTM	LSTM	SVM
RMSE (demand)	0.02	0.06	0.10
MAE (demand)	0.1	1.4	0.95
RMSE (price)	0.04	0.08	0.15
MAE (price)	0.47	1.56	1.09

## References

[1] Wang P., Liu B., Hong T. (2016). Electric load forecasting with recency effect: A big data approach. Int. J. Forecast..

[2] Ludwig N., Feuerriegel S., Neumann D. (2015). Putting Big Data analytics to work: Feature selection for forecasting electricity prices using the LASSO and random forests. J. Decis. Syst..

[3] Lago J., de Ridder F., de Schutter B. (2018). Forecasting spot electricity prices: Deep learning approaches and empirical comparison of traditional algorithms. Appl. Energy.

[4] Wang K., Xu C., Zhang Y., Guo S., Zomaya A. (2017). Robust big data analytics for electricity price forecasting in the SG. IEEE Trans. Big Data.

[5] Haupt S.E., Kosović B. (2017). Variable generation power forecasting as a big data problem. IEEE Trans. Sustain. Energy.

[6] Chen J., Yang Y., Wang Z., Li Y., Guo Y., Cui J., Zhu D. (2017). Distribution fault outage cost evaluation and hotspot area recognition based on big data. J. Eng..

[7] Kenner S., Thaler R., Kucera M., Volbert K., Waas T. (2017). Comparison of SG architectures for monitoring and analyzing power grid data via Modbus and REST. EURASIP J. Embed. Syst..

[8] Tang Y., Yang J. Dynamic event monitoring using unsupervised feature learning towards SG big data. Proceedings of the 2017 International Joint Conference on Neural Networks (IJCNN).

[9] Grolinger K., L’Heureux A., Capretz M.A.M., Seewald L. (2016). Energy forecasting for event venues: Big data and prediction accuracy. Energy Build..

[10] Zhou K., Fu C., Yang S. (2016). Big data driven smart energy management: From big data to big insights. Renew. Sustain. Energy Rev..

[11] Koseleva N., Ropaite G. (2017). Big data in building energy efficiency: Understanding of big data and main challenges. Procedia Eng..

[12] Singh S., Yassine A. (2017). Mining energy consumption behavior patterns for households in SG. IEEE Trans. Emerg. Top. Comput..

[13] Daut M.A.M., Hassan M.Y., Abdullah H., Rahman H.A., Abdullah M.P., Hussin F. (2017). Building electrical energy consumption forecasting analysis using conventional and artificial intelligence methods: A review. Renew. Sustain. Energy Rev..

[14] Kezunovic M., Xie L., Grijalva S. The role of big data in improving power system operation and protection. Proceedings of the 2013 IREP Symposium on Bulk Power System Dynamics and Control-IX Optimization, Security and Control of the Emerging Power Grid (IREP).

[15] Jaradat M., Jarrah M., Bousselham A., Jararweh Y., Al-Ayyoub M. (2015). The internet of energy: Smart sensor networks and big data management for SG. Procedia Comput. Sci..

[16] Mi J., Wang K., Liu B., Ding F., Sun Y., Huang H. A multiobjective evolution algorithm based rule certainty updating strategy in big data environment. Proceedings of the GLOBECOM 2017—2017 IEEE Global Communications Conference.

[17] Xu C., Wang K., Li P., Xia R., Guo S., Guo M. (2018). Renewable Energy-Aware Big Data Analytics in Geo-distributed Data Centers with Reinforcement Learning. IEEE Trans. Netw. Sci. Eng..

[18] Moghaddass R., Wang J. (2017). A hierarchical framework for SG anomaly detection using large-scale smart meter data. IEEE Trans. Smart Grid.

[19] Munshi A.A., Yasser A.-I.M. (2017). Big data framework for analytics in SGs. Electr. Power Syst. Res..

[20] Munshi A.A., Mohamed Y.A.I. Cloud-based visual analytics for SGs big data. Proceedings of the 2016 IEEE Power and Energy Society on Innovative Smart Grid Technologies Conference (ISGT).

[21] He X., Ai Q., Qiu R.C., Huang W., Piao L., Liu H. (2017). A big data architecture design for SGs based on random matrix theory. IEEE Trans. Smart Grid.

[22] Hou W., Ning Z., Guo L., Zhang X. (2017). Temporal, Functional and Spatial Big Data Computing Framework for Large-Scale Smart Grid. IEEE Trans. Emerg. Top. Comput..

[23] Alamaniotis M., Gatsis N., Tsoukalas L.H. (2018). Virtual Budget: Integration of electricity load and price anticipation for load morphing in price-directed energy utilization. Electr. Power Syst. Res..

[24] Amjady N., Daraeepour A. (2009). Mixed price and load forecasting of electricity markets by a new iterative prediction method. Electr. Power Syst. Res..

[25] Alamaniotis M., Bargiotas D., Bourbakis N.G., Tsoukalas L.H. (2015). Genetic optimal regression of relevance vector machines for electricity pricing signal forecasting in smart grids. IEEE Trans. Smart Grid.

[26] Zhang Y., Quan Z., Caixin S., Shaolan L., Yuming L., Yang S. (2008). RBF neural network and ANFIS-based short-term load forecasting approach in real-time price environment. IEEE Trans. Power Syst..

[27] Khotanzad A., Zhou E., Elragal H. (2002). A neuro-fuzzy approach to short-term load forecasting in a price-sensitive environment. IEEE Trans. Power Syst..

[28] Rabiya K., Javaid N. A short-term load and price forecasting using optimized LSTM in SG. Proceedings of the IEEE ICC’20—NGNI Symposium.

[29] Zheng J., Xu C., Zhang Z., Li X. Electric load forecasting in smart grids using long-short-term-memory based recurrent neural network. Proceedings of the 2017 51st Annual Conference on Information Sciences and Systems (CISS).

[30] Elia Grid Data. http://www.elia.be/en/grid-data/dashboard.

[31] Cousineau D., Chartier S. (2010). Outliers detection and treatment: A review. Int. J. Psychol. Res..

[32] Hochreiter S., Schmidhuber J. (1997). Long short-term memory. Neural Comput..

[33] Gers F.A., Schmidhuber J., Cummins F. Learning to Forget: Continual Prediction with LSTM. Proceedings of the 9th International Conference on Artificial Neural Networks: ICANN ’99.

[34] Rao R. (2016). Jaya: A simple and new optimization algorithm for solving constrained and unconstrained optimization problems. Int. J. Ind. Eng. Comput..

